# PROTOCOL: What are the effects of different elements of media on radicalization outcomes? A systematic review

**DOI:** 10.1002/cl2.1148

**Published:** 2021-03-05

**Authors:** Michael Wolfowicz, Badi Hasisi, David Weisburd

**Affiliations:** ^1^ Institute of Criminology, Faculty of Law Hebrew University of Jerusalem Jerusalem Israel; ^2^ Department of Criminology, Law and Society George Mason University Fairfax Virginia USA

## Abstract

**Objectives:**

In this systematic review and meta analysis we will collate and synthesize the evidence on media‐effects for radicalization, focusing on both cognitive and behavioral outcomes. The goal is to identify the relative magnitudes of the effects for different mediums, types of content, and elements of human‐media relationships.

**Methodology:**

Random‐effects meta analysis will be used and the results will be rank‐ordered according to the size of the pooled estimates for the different factors. Meta‐regressions, moderator analysis, and sub‐group analyses will be used to investigate sources of heterogeneity.

**Implications:**

The results of this review will provide a better understanding of the relative magnitude of the effects of media‐related factors. This information should help the development of more evidence‐based policies.

## 1 BACKGROUND

### 1.1 The problem, condition or issue

Dissident and terror groups have long used the media as a vehicle for transmitting their grievances, claims, and ideologies to larger populations. Occasionally, exposure to these messages and their associated images may serve to attract new supporters and recruits, which is one of the main objectives of terrorists' use of the media. In order to ensure that they were given air‐time, terrorist groups would have to carry out actions that were deemed sufficiently “newsworthy”. Some have claimed that this led to a competition for air‐time, in which terrorists resorted to increasingly spectacular attacks throughout the 1960s and 1970s that included skyjackings, embassy takeovers, and bombings. By engaging in such actions, terrorists were assured that the media would promote their actions, and in doing so, their grievances and message as well (Bandura, 1990; Nacos, 2006; Scrivens & Conway, 2019). Individuals may first become acquainted with terrorist groups and their grievances through the news media. And the way in which the news media portray different conflicts, such as the wars in Iraq and Afghanistan, and report on casualties, can also serve to increase radicalization in different ways. For example, it can increase an individual's feelings of hate or resentment towards a country involved in the conflict. It can also increase perceived injustice, or collective relative deprivation. Each of these are known risk factors for radicalization (Wolfowicz et al., 2019), and can be influenced by the media.

Already in ancient times, terrorist groups leveraged media to promote their cause, first through graffiti, coins, and posters, and later with the advent of the printing press which they leveraged to distribute propaganda and recruitment materials through pamphlets and literature (Scrivens & Conway, 2019). Groups such as Al‐Qaeda can be seen as being early adopters of new media‐technology, which they have leveraged for communicating propaganda, and connecting with and between supporters and potential recruits. In the 1980s the group took advantage of new audio and video recording technologies to process and distribute what started out as crude recordings. They later progressed to the development of more sophisticated videos, high‐quality magazines, and took up an early presence on the internet already in the early 1990s with websites and members‐only chatrooms/forums (Lieberman & Collins, 2008). Since then, radical groups from left to right have engaged in creating music, alternative radio and television, animations, and video games to encode their messages (Seib, 2008). Terrorist groups and their co‐ideologues have established a solid presence on the most regularly used social‐media sites, including Facebook, Twitter, and YouTube. The dark web has also provided new opportunities for more secretive communications, as have mobile communications such as WhatsApp and Telegram, with the latter having been referred to as ISIS' preferred platform (Weimann, 2016). In the current environment, it is not only terrorist and radical groups who are producing and disseminating radical content. In fact, independent ideologues may be responsible for the majority of the production and dissemination (Holbrook, 2019).

Since at least the 1970s, researchers have examined media effects in the context of a range of deviant cognitions and behaviours, including those pertaining to aggression and violence. While the literature has found such positive relationships to exist, the evidence highlights differential effects between different forms of media, and different types of content. For example, exposure to violent fiction and horror movies, television news, the Internet, and video games, have been found to have differential effects on increasing aggression and analogous outcomes (Huesmann & Taylor, 2006). But despite this rich literature, there is little knowledge about the magnitude of the effects of different types of media on radicalization, a specific type of deviant outcome with both cognitive and behavioural dimensions. That is, we do not actually know if television, Internet, and video games increase the likelihood of radicalization, to what degree, and what the differential effects of these different media are. In the absence of systematic investigation and synthesis addressing such issues there is little evidence in which to guide policy and decision making in the area of media regulation.

### 1.2 The intervention

Radicalization has become somewhat of a catch‐phrase in recent years but is generally used to refer to the developmental process through which individuals pass on the way to engaging in acts of radical violence, such as terrorism. However, researchers and policy‐makers alike have increasingly come to adopt a more nuanced approach, whereby radicalization denotes the adoption of certain ideas, opinions, beliefs and attitudes that *could* underpin eventual acts of terrorism (McCauley & Moskalenko, 2017). As such, there are actually two distinct, albeit interrelated outcomes of radicalization, namely the cognitive and behavioural outcomes), analogous to the distinctions between criminal cognitions and criminal behaviours (Wolfowicz et al., 2020). Cognitive radicalization relates to the justification of acts of radical violence in defence of a cause or ideology. This could mean violent attacks against property or persons of a sub‐terroristic nature, or attacks against persons or property that are considered terrorism offences according to the law of the countries in which they occur. For a small number of individuals who hold such radical attitudes, they may develop a sense of a personal obligation to engage in radical violence themselves, and they may express, or otherwise admit to such intentions or willingness (Leuprecht et al., 2010). However, even for those expressing such intentions, only a small minority (less than 1%) will actually ever go on to actually to behavioural radicalization and engage in acts of violence in defence of a cause or ideology (McCauley & Moskalenko, 2017).

While it is true that not all individuals who engage in radical behaviours are necessarily the most radicalized in the cognitive sense, cognitive radicalization is almost always a necessary precursor to behavioural radicalization. For this reason, it is important to examine cognitive and behavioural radicalization as two distinct, albeit interrelated outcomes of radicalization. One of the main goals of radicalization research is to identify why some members of an aggrieved group develop radical attitudes, whilst others do not, and why some of those who hold radical attitudes will go on to engage in radical behaviours, while most will not. It has been suggested that the cumulative and interactive weight of risk factors serve to explain a large portion of the variance (Stern, 2016). Exposure to, or the presence of a risk or protective factor in an individual's background, such as being a victim of discrimination, or conversely strong social bonds, increases or decreases the likelihood respectively that they will exhibit one of the outcomes of radicalization (Wolfowicz et al., 2020). One of the most frequently mentioned putative risk factors is exposure to media, or at least certain types of media, with the internet being the medium most frequently cited (Gill et al., 2017). However, the internet is not the only form of media that could potentially increase the risk of radicalization by being used as vehicles for the spreading of radicalizing content. Other forms of both “old” and “new” media may include; television, radio, music, print media, social media, and video games. Additionally, beyond mere exposure, there are other dimensions to human‐media interactions such as frequency, duration, and intensity of exposure. Each of these different mediums and dynamics have the potential to increase the likelihood that an individual will come to develop radical attitudes, and, or engage in radical behaviours. Unfortunately, little is known about the differential effects that these potential sources of risk may carry when it comes to radicalization (Scrivens et al., 2020).

Traditionally, television has been said to be one of the primary platforms that terrorists use to radicalize new supporters and potential recruits (Bandura, 1990). In fact, it has been argued that increasingly brazen or “spectacular” terrorist attacks are often concocted with newsworthiness in mind, as groups vie for air‐time and attention (Weimann & Brosius, 1991). As noted above, terrorist groups hope to attract new supporters and recruits by keeping their messages, grievances, and images in front of a wide audience. Some studies have found that as terror attacks receive more television coverage, interest in the groups on the internet increases (Fantazzini et al., 2020; Jetter, 2019). But the interplay between news and broadcasting can also potentially contribute to radicalization in other ways. For example, traditional and conservative populations in places such as the Middle‐East may be exposed to images of a materialistic western (Mousseau, 2011), or selective broadcasts of counter‐terrorism and war‐related operations, that may increase hostility towards the west. Satellite television has enabled radical preachers (including right‐wing extremists) to directly broadcast their sermons into the homes of millions. Each of the different types of television consumption may have differential effects on radicalization (Matusitz, 2013).

In some places in the world, such as Africa, radio still remains a widely used source for news, and entertainment, and it plays an important role in propagating support for different radical ideologies and groups (Hackett & Soares, 2015). One of the few evaluation studies to be conducted in the counter‐radicalization literature was of a peace‐radio program in Africa. The study found lower levels of radicalization in areas in which the programming was instituted compared to the areas used for the control group (Aldrich, 2014; Marrone et al., 2020). In other places, relatively new forms of digital talk radio, and podcasts, have been taken up by commentators, analysts, and other personalities offering more specialized programming that focuses on specific topics. Radio and podcasts, especially those dealing with conspiracy theories, have often been referred to as a source of radicalization for the far right in particular. Arguably the most popular conspiracy stations, Alex Jones' Info Wars, which started as a radio program, has been implicated in having directly influenced at least two right‐wing terror attacks in the United States in recent years (Hamm & Spaaij, 2017). Audio of recorded sermons was one of Al‐Qaeda's original propaganda tools. In a study of 57 convicted terrorists from the United Kingdom, audio recordings represented the bulk of radical content found in their possession, even up until 2017 (Holbrook, 2019).

The role of music has also figured prominently in the radicalization literature, especially in research on right‐wing radicalization. A study of former white‐supremacists revealed that the formers felt that the music played an integral role in shaping and sustaining their extremist ideologies (Simi et al., 2017). Music has also been used by Islamist groups to draw new supporters and recruits, including most recently by ISIS. Music is known to effect a range of cognitive and behavioural outcomes (Timmerman et al., 2008), including with regards to radicalization (Baier et al., 2019). Music containing violent lyrics has been found to increase hostility and aggression (Andersen et al., 2003), whereas different genres, such as rap and heavy metal, and white‐power music, have been found to have differential effects on analogous outcomes (LaMarre et al., 2012).

Despite the move to online sources for music, television, and radio, print media still remains an important source for news and for conveying different ideological positions. In Pakistan, the ideological slant local newspapers have given to the portrayal of drone strikes on terrorist targets has been referred to as a “major source” of continued radicalization (Raza & Awan, 2013). Across the Middle‐East, and even in places such as downtown London, books such as Adolf Hitler's *Mein Kampf*, and the fictional *Protocols of the Edlers of Zion* are sold alongside more recent radical publications (Patterson, 2015 Phillips, 2006). The 1978 publication *The Turner Diaries* is a “must‐read” in white‐nationalist circles, and has been directly linked to a number of high‐profile terrorist incidents and groups, in the United States and Europe, including the Oklahoma City Bombings (Berger, 2016). Other publications, such as *The Camp of the Saints* have been said to be popular among right‐wing extremists and serve to foment racist attitudes.

Finally, the Internet, which has received the bulk of the attention of researchers in recent years, provides an environment that is especially conducive to radicalization for a number of reasons. For example, the Internet is a space lacking in oversight, regulation, and policing when it comes to things like hate‐speech and radicalizing content. It is not difficult for an individual to seek out and find the latest edition of Al‐Qaeda's *Inspire* or ISIS' *Dabiq* (online magazines), beheading videos (such as the famous Daniel Pearl video) (Frissen, 2020), or the manifesto of Andres Bravik. Similarly, the internet also makes it is easier for individuals to find views and opinions that are less accepted in general society, and to connect with like‐minded individuals and groups who share such views and opinions. In addition to availability, the internet also provides for more instant and frequent access to such content and groups (Pauwels & Schils, 2016). As a result, individuals can immerse themselves in networks and communities that provide reinforcement for deviant attitudes and alternative forms of social control. In some cases, and individual's attachment to their online associations and networks can be just as strong, and even stronger than for their offline associations (Neumann, 2013). Additionally, when an individual becomes immersed in an online environment in which certain attitudes are skewed toward a particular stance vis‐à‐vis an issue or behaviour, then the network can act as an “echo chamber”, promoting confirmation bias, polarization, and opinion extremism (Sunstein, 2009).

### 1.3 How the intervention might work

The way in which exposure to or consumption of media is theorized to influence radicalization is not some sort of “silver bullet” or “hypodermic needle”. That is, simple exposure to radical content does not suddenly or singularly cause an individual to justify ideologically motivated violence, support a specific group or seek to carry out an act of radical violence (Baines & O'Shaughnessy, 2019). Experimental research has found that mere exposure to radical content or messages has little to no effect on changes in radical attitudes (e.g., Rieger et al., 2017; Shortland et al., 2017). Rather, as highlighted by a number of theoretical perspectives, there are different mechanisms by which the repeated exposure to messages highlighting the grievances or actions of a group, and/or which justify the use of violence in furtherance of a group or ideology's objectives, contribute to the development of radical attitudes, and which could underpin radical behaviours (Baines & O'Shaughnessy, 2019).

Arguably the most important framework for understanding the media‐deviance nexus is Albert Bandura's (1971) Social Learning Theory (SLT), and his extension of it, Social Cognitive Theory (SCT). First applied to understanding the effects of violent television on aggression, SCT holds that the repeated exposure to messages and images relating to terrorism may lead to moral disengagement, and ultimately radicalization. For example, by emphasizing the “bad deeds” of the victim, such as their role in forms of oppression against other groups (e.g., the U.S.' drone attacks in Afghanistan and Pakistan), it can be suggested that they were or are deserving of violence, that they “had it coming to them”, or that the violence is insignificant in light of their own actions. Such emphasis therefore dehumanizes the target of terrorism, whilst legitimatizing the perpetrators, or at least their actions (Bandura, 1990). More generally speaking, the repeated exposure to messages and images of violence can lead to “Emotional desensitization”, which can contribute to radicalization since it makes it easier to justify or support the use of violence (Neumann, 2013, p. 435). It is also possible that an individual may come to feel a sense of vicarious group‐based injustice or strain by exposure to images or messages of the plight of a particular group with whom they identify (Agnew, 2010, 2016; Nivette et al., 2017).

Relatedly, social learning views the adoption of deviant attitudes, cognitions, and behaviours as being learnt in the same way as normative attitudes, cognitions, and behaviours. Whether deviant or normative, learning occurs primarily through differential associations, such as peers, family, network members, and media, to whom the individual attaches differing degrees of importance or legitimacy. Differential associations, among which media can take a central role, provide the individual with a balance of definitions for or against a particular attitude or behaviour. When the balance of definitions in favour of the attitude or behaviour outweigh those against it, there is an increased likelihood that the individual will adopt the attitude or behaviour (Akers & Silverman, 2004; Pauwels & Schils, 2016). Research has found that having engaged with other extremists online increases the likelihood that an individual with hold radical attitudes, and also engage in radical behaviours (e.g., Pauwels & Schils, 2016). Differential associations also provide models for imitation, and sources of differential reinforcement, in which the individual learns about what sort of social response they can expect to receive from adopting the attitude or engaging in the behaviour (Akers, 1998).

But the social learning and cognitive perspectives also describe the effects of media in terms of reciprocal determinism, whereby pre‐existing biases shape and determine media consumption, and that consumption further reinforces those prior beliefs (Huesmann & Taylor, 2006). This type of media effect has been described as a reinforcing spiral (Slater, 2007). Indeed, as per the accounts of many former extremists, they did not simply stumble upon radical content and groups online but rather actively sought it out (Gaudette et al., 2020). Recent research has found that the consumption of radical content actively sought out by a user has a greater effect on increasing radicalization than the consumption of radical content by a user who merely came across it (Frissen, 2020).

For a number of criminological theories, including both social control and self‐control perspectives, the role of self‐selection plays a central role. Self‐selection refers to the decisions the individual makes about with whom they will associate, and specifically with respect to the active seeking out of associations with certain types of individuals, groups or networks, often like‐minded or similar to themselves. The tendency for self‐selection with similar associations or ideologically congruent media can lead to the creation of the above‐noted media echo‐chamber, in which an individual becomes immersed within content and networks that reinforce existing attitudinal tendencies, and block out opposing messages. Echo chambers are characterized by their high level of insularity, and isolation from external networks, providing a prime criminogenic environment in which social learning of deviant attitudes and behaviours, such as radicalization can occur (Ramakrishna, 2010; Saddiq, 2010; Stevens & Neumann, 2009; Sunstein, 2009; Von Behr et al., 2013; Warner, 2010; Wojcieszak, 2009, 2010). Research has found that having stronger ties and connections with online networks increases the likelihood of radical attitudes (e.g., Ellis et al., 2016; Wojcieszak, 2010), especially with regard to highly similar ties (Wojcieszak, 2009).

Echo chambers also increase the likelihood that network members' identities will become “fused” with a group‐based identity. Former extremists seem to corroborate this perspective, having reported that during their radicalization they found it important to become part of, and accepted as a member of an ideologically homogenous group, and that this membership came to form an important part of their identity (Gaudette et al., 2020). Group‐based identities are known risk factors for radicalization (Wolfowicz et al., 2020). While echo chambers exist offline, the effects of online echo chambers are believed to be stronger, since the online environment provides for more frequent access, and therefore greater immersion in the network (Klausen et al., 2020).

It is also important to consider the way in which mass‐media operates and affects individuals' perceptions of issues. According to the “agenda setting theory” (ATS) the repeated discussion of a particular topic in the media serves to draw the consumer's attention to the topic and issue by emphasizing that it is something they ought to care about (McCombs, 2004). As noted above, an individual may first become acquainted with a terrorist group and their grievances through their exposure to news coverage of an attack. A recent study found that Al‐Qaeda attacks receiving more news coverage by mainstream media led to a significant increase in searches for the group and their propaganda materials online (Jetter, 2019). Related to ATS, “priming theory” suggests that the media presents a narrative surrounding the topic or issue that suggests to the consumer how they ought to feel about it (Iyenger & Kinder, 1987; McCombs & Shaw, 1972; Williams, 2003). Here, even if the media are not necessarily being supportive of the terrorist groups, highlighting the victims of counter‐terrorism responses can lead to increased identification with a radical ideology (Crocq, 2007).

In this regard, media such as television and the internet also work to develop narratives around certain issues. Mainstream media “plant” information within the individual, which can have significant effects on the way reality is evaluated, importantly with regard to the way which we evaluate phenomena, such as perceived increases or decreases in certain types of crime (Shrum, 1995). As such, the media can affect the number of victims an individual perceives to have been created at the hands of a military airstrike. This could lead to increasing hate of a government, and/or increasing identification with the grievances espoused by a terrorists group, both of which can contribute to radicalization.

The power of the media to alter identities in such a way is certainly not limited to the internet or television. With regards to print media, Copeland (2019) highlights how narratives and stories have the power to engender “transportation”, in which the reader becomes immersed and absorbed in the world of the narrative. According to the theory, a “traveller” is one who traverses the pages of a piece of content (although it can also be a movie or other piece of media) that causes an emotional reaction in the traveller in which the narrative of the content is applied to the traveller's real world. This causes them to see the world around them through a new lens, the lens of the narrative. Copeland (2019) cites other studies that have found evidence of “transportation” in the biographical accounts of former terrorists from a range of ideologies. Books such as the *Turner Diaries* may cause an individual to being to see the world through a new, radicalized lens.

There are clearly numerous ways in which different types of media may ultimately contribute to an individual developing radical attitudes, and in some instances engaging in acts of radical violence. While this short description should not be considered a comprehensive review of the different perspectives, it highlights how the different types of media have the potential to contribute to radicalization. An extensive literature distantly related to the topic of the current review has found that exposure to images related to terrorism, such as those depicted in the media, can have negative psychological effects (e.g., Felix et al., 2020; Hurtado‐Parrado et al., 2020). However, it remains under‐researched as to how the different types of exposure and interactions with different medias impact radical attitudes and behaviours specifically. In line with what is known about risk and protective factors for radicalization, it can be expected that the cumulative and interactive effects of exposure to different types of media, both old and new forms, increases or decreases the likelihood of radicalization depending on the nature of the exposure (Scrivens & Conway, 2019; Wolfowicz et al., 2020).

### 1.4 Why it is important to do the review

There are a number of reasons why it is important to do this review. First, there is a debate in the literature as to the extent to which the media actually plays a role in radicalization. While many view the media and the internet in particular as one of the most important factors in radicalization, others dismiss it as have little or no effect at all (e.g., Benson, 2014). Some are even of the opinion that radical content and spaces can provide a non‐violent outlet for the voicing of grievances, and an opportunity for radicals to feel a sense of contribution without the need to resort to violent behaviours offline (Barbera, 2014; Helmus et al., 2013; Kardas & Özdemir, 2018). While some studies have found that the media (and the internet in particular) has played a role in the radicalization of most western terrorists in the last decade (e.g., Gill et al., 2017), a recent study reporting on in‐depth interviews with dozens of Islamist prisoners found that almost all felt they had not been influenced by the media at all (Baugut & Neumann, 2020). Recently, some scholars have called for the need to integrate radicalization research within the broader and more well‐developed literature on media effects and deviance. In doing so, research should draw on the theoretical and methodological frameworks from this literature. This includes, among other things, attempting to isolate the relative effects of different types of media by comparing them with each other (Scrivens et al., 2020). As such, a systematic review of the effects of media‐related factors on radicalization can hopefully bring some degree of reconciliation to the debate.

Second, most western governments do see the media, and the internet in particular, as a potential source of radicalization and have instituted different policies and regulations that seek to curb the radicalizing effects. One approach has been to “take down” as much radical content as possible. But Neumann (2013) is of the opinion that the “take‐down” tactic is “the least desirable” approach to dealing with online radicalization. One of the reasons is that “take downs” inevitably lead to impingements on free speech. Moreover, radical groups may claim take‐downs as evidence that the “enemy” does not really believe in free speech, or is discriminatory against their in‐group. As a result, take‐downs can have a backlash effect by increasing grievances, or serving as proof for the claims of radical groups (Weirman & Alexander, 2020). This case is emblematic of a common criticism of counter‐radicalization policies, namely that “poorly designed programs are not only a waste of resources but also may increase the risk of violence” (Koehler, 2019). Specifically, the lack of evidence‐based policy “has made way for programming that has either been overly broad or inappropriately targeted, resulting in ineffectiveness, or an exacerbation of existing tensions” (Harper, 2018, p. 23). This systematic review aims to be able to identify evidence that could help support the development of more evidence‐based policies.

To date, there is little in the way of systematic investigation into the role of different media‐related factors and radicalization that could help to provide evidence for the two sides of argumentation. In a recent systematic review and meta‐analysis, Wolfowicz et al. (2020) identified passive and active forms of media exposure as risk factors for attitudinal radicalization among dozens of other factors. Passive exposure relates to consuming content through reading, listening or watching, whereas active exposure relates to engaging with content through producing, disseminating, discussing, or interacting with it. The general finding was that while each presented risk, the magnitudes of the effects were considerably smaller than other factors, including those related to offline associations. However, as per the above discussion, this level of aggregation (passive and active) may conceal a deeper understanding of the differential effects. Additionally, the review was focussed on risk factors more generally and used strict inclusion criteria, leading to the possibility that important studies were missing from the analysis.

The only other related review that we have identified, conducted by Hassan et al. (2018). One of the primary criticisms that can be levelled against this study is that its inclusion criteria were far too broad to enable a meaningful quantitative synthesis. Perhaps as a result of casting the net so wide, the Hassan et al. (2018) review also missed a number of important studies that directly measure the effects of media exposure on individuals' radical attitudes and behaviours (Scrivens et al., 2020).

## 2 OBJECTIVES

The primary objectives of this systematic review are to provide quantitative information that can help in answering important questions regarding the risk or protective effects of media‐related factors associated with radicalization outcomes. As a field‐wide review, the first objective is to identify what the different media‐level factors are, without any predeterminations. We also seek to identify whether the same factors figure for different radicalization outcomes, namely cognitive and behavioural radicalization. We hypothesize that we will identify a greater range of factors beyond simple passive exposure to different media platforms. In line with previous work (Lösel et al., 2018; Wolfowicz et al., 2019), we also expect to identify a larger range of factors and more data will be found for outcomes associated with cognitive radicalization. The second objective is to categorize, organize, and arrange the factors in a series of rank orders according to their identified effect sizes in order to assess the absolute and relative importance of the different factors. In doing so, we may be able to aggregate the factors in order to identify the relative differences in the effects between “old” and “new” medias (such as books and newspapers vs. social media), as well as passive and active forms of consumption. This may help to also identify differences between online and offline sources of risk for radicalization. Lastly, we will attempt to identify how factors differ across regions (e.g., United States and the EU) and ideological doctrines (e.g., Right‐wing, left‐wing, religious, etc.). As such, through the use of meta‐analytic techniques this review seeks to address the following questions:

1. What are the different media‐related factors for cognitive radicalization?
2. What are the different media‐related factors for behavioural radicalization?
3. What are the relative magnitudes of the effect sizes for the different factors across the different outcomes?
4. What are the sources of heterogeneity between factors as they pertain to different ideologies and radical doctrines (e.g. right‐wing and Islamist inspired), and to different regions (e.g., EU and the United States)?

## 3 METHODS

### 3.1 Criteria for considering studies for this review

#### 3.1.1 Types of studies

The review seeks to identify, collate, and synthesize observational studies, namely cross‐sectional, longitudinal, and case‐control designs. The outcomes from these studies are expected to be derived primarily from self‐reports. However, the review will also include studies based on clinical reports (e.g., practitioner coded data), administrative data (e.g., from security services), and secondary databases constructed from multiple open sources (e.g., Profiles of Individual Radicalization in the United States [PIRUS]).

Experimental studies will be included when the experimental manipulation involves some variation (e.g., at least two conditions) of some form of human‐media interaction, such as exposure to news media, videos. That is, the experimental condition will differ in terms of the type of media that participants are exposure to, or the contents of the media that participants are exposed to in order to assess differences in one of the eligible outcomes. This includes lab‐based experiments, internet‐based experiments, and experiments using vignette designs. For these types of studies to be included the treatment or control must include at least one condition in which the exposure is theorized to increase the likelihood of radicalization, so as to distinguish the studies and this review from research (including systematic review) on counter‐narratives (e.g., Carthy et al., 2020). While these studies' outcomes are also expected to be derived primarily from self‐reports but may also include clinical reports (e.g., practitioner coded data). We will include all such studies irrespective of the method of assignment (e.g., random, random‐blocked).

For all study types, there must be variation on the dependent variable, (e.g., a proportion of the sample displays, or does not display the outcome of interest in the case of dichotomous measures, or displays it to varying degrees in the case of ordinal, discrete or continuous measures).

For cross‐sectional studies, we will include studies irrespective of the nature of the sample with respect to sampling. That is, we will include studies using any variety of sampling methods, such as (but not limited to): Random, Representative, Quota, Convenience, Purposive, Snowball, etc.

For case‐control studies, we will include both prospective and retrospective studies, however, based on our familiarity with the wider literature we do not expect that any prospective studies exist. In both cases, we expect that the “case” sample is made up of a group of individuals who display one or more of the outcomes of interest (see Section 3.1.4) as assessed by self‐reports, clinical reports, or administrative decisions (such as a clear violation of the law in line with the outcomes of interest described below). We expect that for these studies, the control sample will be made up of one of the following types of individuals:

1. Individuals assessed for or who were suspected of having displayed one of the outcomes of interest but who were found not to, or who were found to display it to a lesser degree than the “case” sample or some chosen threshold
2. Individuals from the general population not displaying the outcome of interest or displaying the outcome of interest to varying degrees that is representative of the natural distribution in the general population.
3. Individuals not displaying the outcome of interest but who display a related, excluded outcome (see Section 3.1.4).

For these studies, controls may be chosen based on having been part of a single cohort (as in the case of prospective studies), or derived from a relevant data set (such as administrative records). However, we will also include studies that match controls from a different data set either based on relevance, overall similarly, or on individual characteristics, as in the case of retrospective studies. We will also include studies whose control sample is derived from the general population and includes individuals considered to be at risk for displaying the outcome of interest specifically, or a normal distribution of individuals displaying or not displaying (or displaying to varying degrees) the outcome of interest.

For longitudinal studies, we will include studies whether they are prospective or retrospective designs, as well as whether they are cohort or panel studies, that measure the indicator and outcome of interest at least two different time points. We set no limits on the time between measurement but expect it to be a period of at least a few months in order for a study to be considered to be a true longitudinal design. For cohort studies, whether they are prospective or retrospective, we expect that the cohort will have some shared characteristic, such as being from a specific age group, geographic setting, or social setting at the time of selection. For panel studies, whether prospective or retrospective, we will include studies irrespective of their sampling (e.g., Random, Representative, etc.).

Observational studies and experimental studies will be treated separately in the analysis.

#### 3.1.2 Types of participants

Previous reviews of the literature have highlighted the dearth of research concerning the media‐radicalization relationship at the individual level. It has been found that the majority of the research focuses on the internet, and specifically on platforms, content, groups and networks. Whilst important, this type of research represents a distinct line of inquiry and is better described as the analysis of “online extremism” rather than “radicalization” (Odag et al., 2019). This type of research does not provide the type of information—due to the units of analysis—that can inform us about the media effects on radicalization (Winter et al., 2020).

As such, given that previous reviews have already dealt with this distinct line of inquiry, the current review seeks to address the gap in the literature concerning the individual level. The review will therefore include studies in which the participants, or units of analysis are individuals. The review therefore excludes studies where the unit of analysis is some form of media content, networks, or groups, or other units of analysis that are unable to provide information pertaining to this review's research questions and objectives.

The review places no restrictions on inclusion based on the individual characteristics of the participants. The review will include all samples regardless of the participants' age, gender, ethnicities, religion, or the type of radicalizing doctrine being investigated (e.g., right‐wing, left‐wing, religious, etc.). Some studies may report on conceptually distinct participants based on age, gender, ethnic/religious group, or the type of ideology being examined (e.g., right‐wing, left‐wing, religious, etc.). Previous systematic reviews have found that such factors rarely impact the pooled estimates in meta‐analyses of indicators pertaining to the same radicalization outcomes taken up by the current review (Wolfowicz et al., 2020). As such, while we will combine studies examining the same indicators into a single analysis regardless of conceptual differences between the participants but will endeavour to explore participant population factors as possible sources of heterogeneity through the use of meta‐regression, and where appropriate moderator analysis will be carried out (see Section 3.3.8).

#### 3.1.3 Types of interventions

The review will include studies that examine at least one media‐related factor as an independent variable that provides an indication of correlation with one of the outcomes of interest. A media‐related factor is considered to be an exposure‐based indicator whose source is in some form of media. For example, a known risk factor for radicalization is exposure to violence (Wolfowicz et al., 2020). This review would consider in‐person exposure to violence to be excluded, but mediated exposure to violence (through television, social media, or other forms of media) would be an eligible indicator. In line with the review's objectives and research questions, the review makes no predeterminations as to what media‐related factors may exist. Nevertheless, in Table 1 we provide some examples of the types of factors that are expected to be identified.

**Table 1:** Examples of types of factors expected

Variable	Description
Passive consumption	Watching/reading/listening of mediated content
Active consumption	Posting/creating/engaging in mediated content
Frequency of use	Television/radio/music/print media/Internet/social media
Network characteristics	Individual (ego) network size/tie‐strength/density etc.
Differential associations	Online deviant peers/network members
User‐level behaviours	Likes/comments/shares/types of posts

#### 3.1.4 Types of outcomes

In line with the literature and previous systematic reviews (e.g., Wolfowicz et al., 2019), this review examines two distinct, albeit interrelated outcomes, namely the cognitive and behavioural outcomes of radicalization. In order to ensure that the review includes studies with comparable outcomes, the review will limit its inclusion to studies whose dependent variables are in line with the outcomes of McCauley and Moskalenko's (2017) Two‐Pyramid Model of radicalization (TPM). The TPM was selected because rather than being a traditional process models of radicalization, it is actually an outcome‐based typological model. The TPM's typologies provide a clear set of criteria for determining if an outcome falls in to one of its categories, as well as for the categorization of the outcome (Wolfowicz et al., 2020). Moreover, when studying related cognitive and behavioural outcomes in parallel, the cognitive outcome should have a high level of specificity with reference to the behavioural outcome (Fishbein & Ajzen, 1975). This criterion is met by the TPM's typologies as described below.

In line with the TPM, studies will be classified as examining either cognitive or behavioural radicalization outcomes, with each of these outcomes including two sub‐categories. With respect to cognitive radicalization this review will include studies that examine radical attitudes or intentions. Studies examining radical attitudes will be included when the dependent variable assesses support for, or justification of the use of radical violence, which is the use of violence towards persons in the name of a cause. This definition strikes a good balance between specificity and sensitivity, leaving room for the inclusion of violence motivated by a range of ideologies, and excluding non‐ideological forms of violence. Studies examining radical intentions will be included when the dependent variable assesses intentions towards engaging in, or an expression of a willingness or desire towards the commission of radical violence. In both cases, studies may use a variety of single or multiple item instruments that include specific references to radical violence generally, or specific instances of radical violence. While the review places no specific restrictions on which measures are used, all measures must include an explicit reference to the use of some form of radical violence in the name of a cause. Some examples of the expected measures for cognitive radicalization outcomes are displayed in Table 2.

**Table 2:** Examples of measures of cognitive radicalization

Measure	Description	Example of usage
*Validated instruments*		
Sympathy for violent radicalization and terrorism (SyFor) (Bhui et al., 2014a, 2014b)	A 16‐item tool measuring sympathies and condemnations of radical behaviours from violent protest to suicide bombings	Frissen (2020)
Activism‐Radicalism‐Intentions Scales (ARIS) (Moskalenko & McCauley, 2009)	10‐item tool assessing intentions to engage in legal and illegal forms of activism	Decker and Pyrooz (2019)
Pro‐violence and Illegal Acts in Relation to Extremism Scale (PIARES) (Ozer & Bertelsen, 2018)	A 6‐item tool using a 7‐point Likert scale	Ozer (2020)
Militant‐Extremist Mindset (MEM) pro‐violence subscale (Stankov et al., 2010)	A 10‐item subscale using a 5‐point Likert scale	Vergani et al. (2019)
*Single measures*		
Some people think that suicide bombing and other forms of violence against civilian targets are justified in order to defend Islam from its enemies. Other people believe that, no matter what the reason, this kind of violence is never justified. Do you personally feel that this kind of violence is often justified to defend Islam, sometimes justified, rarely justified, or never justified?	4‐point scale: 1 = *often*, 4 = *never*	PEW report (see McCauley, 2012; Victoroff et al., 2012).
What is your attitude towards the murder of Theo van Gogh, in November 2004?	5‐point scale: 1 = *very negative*, 5 = *very positive*	Doosje et al. (2013)
‘Some people said that the July (7/7/) bombings were justified because of British support for the U.S. war on terror. Do you agree?	4‐point scale: 1 = *strongly disagree*, 4 = *strongly agree*	Tausch et al. (2009)

While some reviews examining a broad, and large number of studies have previously assessed the relationship between individual factors radical attitudes and intentions separately (Wolfowicz et al., 2020), others have combined them as a single measure of cognitive radicalization (Emmelkamp et al., 2020). Given the high degree of similarity in measurements used, and the fact that we do not expect a large number of studies as has been found in larger reviews (e.g., Wolfowicz et al., 2020), we will group studies examining radical attitudes and intentions together as a single outcome for cognitive radicalization. In a situation in which a study reports on two separate outcomes for cognitive radicalization (e.g., radical attitudes and radical intentions), two separate analyses will be conducted in which alternative effect sizes will be used. Alternatively, if two or more studies contributing to a particular analysis report on two or more outcomes, subgroup analysis will be conducted. Where the analysis for any particular factor includes effect sizes derived from at least two studies for each of the outcome categories (attitudes and intentions), moderator analysis will be performed in order to assess the degree of between‐outcome measurement heterogeneity (see Section 3.3.12).

With respect to behavioural radicalization, this review will include studies that examine engagement in radical behaviours, which are sub‐terroristic forms of illegal, radical violence (so as to be distinguished from legal, non‐violent forms of activism), as well as studies that examine involvement in terrorism. With regard to radical violence, the review will include studies that assess self‐reports of prior engagements in radical violence (e.g., Pauwels & Schils, 2016), as well as studies based on administrative data, or databases derived from both official and open sources. With regard to terrorism, the review considers terrorism to be any form of planned or successful terrorism offending as defined by the law of the country in which the offence occurred. The review will include studies whose samples are drawn from data from official and open sources (e.g., PIRUS). Studies examining both self‐reported forms of radical violence and terrorism involvement will be combined as a single outcome of behavioural radicalization. When there are more than two studies from each of these categories in a single analysis, moderator analysis will be used to assess whether any between‐outcome measurement heterogeneity exists (see Section 3.3.12).

#### 3.1.5 Duration of follow‐up

While no limitations will be placed on the duration of follow‐up, longitudinal studies will be grouped together by the time interval to follow up. We will consider the length of time between two time points from 0 to 12 months as a short‐term follow‐up period, 12 to 24 months as a mid‐term follow‐up period, and 24> months as a long‐term follow‐up period. A separate analysis will be conducted for each of the time points that includes studies whose follow‐up period falls within each of the intervals and, or who present separate results for different time points that fall into more than one of these intervals.

#### 3.1.6 Types of settings

No limitations will be placed on the settings from which the samples of studies originate. Where possible, subgroup, meta‐regression or moderator analysis will be used to identify differential effects for different regional settings (see below).

### 3.2 Search methods for identification of studies

Searches will be carried out to identify both published and unpublished studies and reports. While primary searches will be conducted in English, which is the primary language for indexing and abstracts, studies identified in other languages will also be assessed for inclusion.

#### 3.2.1 Search terms

Given that we are interested in identifying what the different media‐related factors for radicalization are, without making any predeterminations (research question #1), the search terms exclude specific interventions/indicators from the search terms (e.g., Internet, social media, television, radio, print media, etc.). In place of this we have included two general terms to capture the types of indicators we are interested in, “media” and “technology”. We have also included the terms “Internet” and “online” in order to account for studies that may refer to these types of medias specifically without reference to media more generally. Additionally, our search terms seek to include the most commonly employed terminology used to refer to radicalization, our problem and outcome of interest. In order to limit our results as much as possible to quantitative studies, we have included a number of search terms that denote study type or methodological approach, with search terms being inclusive for both observational and experimental studies.

Standard search string: Abstract/Title = (Radical* OR Extrem* OR Terror* OR Action OR Politically motivated* OR Ideological*) AND (Media OR Technology OR Internet OR Online) AND (Quantitative OR Empirical OR Survey OR Regression OR Multivariate OR Correlation OR Experiment* OR Manipulat* OR coefficient* OR covariat* OR longitudinal OR paramet* OR predict* OR questionnaire* OR sampl* OR standard deviation*, OR statistic* OR variable* OR variance)

#### 3.2.2 Electronic searches

All searches will be carried out (where possible) on both titles and abstracts jointly. Where possible, searches will be expanded to encompass indexed keywords. The search string may be shortened or limited in cases where limitations on search string length are imposed by the different databases. Given that the topic of radicalization is known to be addressed by a range of disciplines (Wolfowicz et al., 2020), searches will be carried out on a number of different databases.

#### 3.2.3 Searching other sources

An initial search will be carried out on the Campbell Collaboration and Cochrane libraries. We will also carry out searches to identify existing reviews and screen their reference lists to identify possible studies that meet the inclusion criteria. During the course of the time when the searches are being carried out, we will contact a number of recognized experts (researchers and organizations) and present them with our review topic, criteria, and initial results, with a request that they direct us to any missing studies that they may be familiar with that could meet the inclusion criteria. For included studies, we will also review their literature reviews and reference lists in order to identify whether they include any additional studies that may meet the inclusion criteria.

In addition to ensuring that the databases in which searches will be performed cover the main fields responsible for the production of research in the field of interest (Wolfowicz et al., 2020), the selection of databases also seeks to consider grey literature (Open Dissertations), and topic specific inquiries (International Security & Counter‐Terrorism Reference Center) (Table 3).

**Table 3:** Databases where searches will be carried out

Database	Access service
ERIC	EBSCO
Criminal Justice Abstracts	EBSCO
Political Science Complete	EBSCO
Violence and Abuse Abstracts	EBSCO
International Security & Counter‐Terrorism Reference Center	EBSCO
Open Dissertations	EBSCO
IPSA (International Political Science Abstracts)	EBSCO
ISI (Social Science Citation Index)	Web of Science
PsycINFO	APA
PubMed	PubMed

Searches and results will be managed and documented in Endnote X9. The results of each search will be stored in a separate file that will include the data of the search, the database where the search was performed, the search syntax used, and the search results.

### 3.3 Data collection and analysis

#### 3.3.1 Description of methods used in primary research

Included studies that examine cognitive radicalization are most likely to employ survey‐based research methods. These methods are most commonly used in cross‐sectional studies and examine exposure to indicators retroactively or concurrently (Wolfowicz et al., 2020). An example of a possible indicator relevant to the current study would be “exposure to terrorist propaganda videos”. A survey item may assess exposure retroactively by asking respondents “Have you ever watched a terrorist propaganda video?”. A similar item could assess concurrent exposure by asking “How often do you watch terrorist propaganda terrorist videos?”.

Included studies that examine behavioural radicalization are most likely to employ secondary data derived from either open‐source of official sources. However, it is also possible that studies will employ survey‐based methods to examine self‐reports of sub‐terroristic radical behaviours. For example, Lieven Pauwels of Ghent University has published a series of papers that include measures of radical content consumption, cognitive radicalization, and self‐reported behavioural radicalization based on a survey of some 6020 youth from Belgium (Pauwels & Schils, 2016).

Included studies are likely to present data and results using a combination of descriptive and inferential statistics. In the case of the former this may include the presentation of group means, proportions, and standard deviations, with or without parametric or non‐parametric forms of null‐hypothesis testing (e.g., *t* tests, *F* tests, *χ* ^2^ tests). In the case of the latter, included studies are likely to present the results of multivariate regression models, including but not limited to OLS, logistic regression, generalized linear models, and different forms of path analysis such as structural equation modelling.

#### 3.3.2 Criteria for determination of independent findings

Given that this review focusses on correlates (risk‐protective factors), it is not expected that a single study will provide multiple measures on a factor from the same domain. Additionally, a separate analysis will be carried out for each of the different factors. Nevertheless, as a rule, a study is unable to contribute more than a single effect size concerning each factor to an individual analysis. In a situation in which a study presents more than single effect size, an internal meta‐analysis will be conducted and its results will be used as the final, singular input. Similarly, in a situation in which two studies are based on the same dataset, and report on the same factor, an internal meta‐analysis will be carried out and the output will be used as the final, singular input. In a situation in which a single study reports on more than one sample, and the results for each sample—or individual study—are presented separately, the results will be treated as independent and will each provide a separate input for the meta‐analysis.

#### 3.3.3 Selection of studies

After searches have been performed, all search results will be downloaded into Endnote X9 and stored in a shared library that all reviewers and research assistants will have access to. A double screening process will be implemented in which two reviewers will screen the titles and abstracts of retrieved items in order to assess whether they relate in any way to the topics of interest. The screening will be performed directly in Endnote, where the reference window will display the titles and abstracts. All studies that are considered to potentially meet the inclusion criteria will be copied to a separate Endnote folder entitled “First screening”. The second stage of this process will include a more thorough reading of the abstracts of selected studies in order to identify whether they are likely to provide quantitative information, and whether they assess an outcome that is in line with the topic of interest. Studies selected at this stage will be copied to a separate Endnote folder entitled “Second screening”. We will identify and download the full‐text PDFs for each of the items in this folder and attach them to their respective references within Endnote. Subsequently, the full‐text PDFs will be used to assess if the study meets the criteria for consideration. This will primarily come from a full reading of the methods section, especially the sections describing the measurement of the outcomes and indicators, as well as the nature of the sample and methods used. Only studies meeting all inclusion criteria will be included in the review and will be copied to a sub‐folder entitled “Final inclusion”. Screening decisions will be made by two reviewers, who will work independently. One of the reviewers is the review's first author. Final inclusion decisions will be compared and any discrepancies will be reconciled in a joint meeting between the reviewers.

#### 3.3.4 Data extraction and management

All coding will be performed by two coders, one of whom is the first author. Coding will be carried out using Excel. At the end of the coding stage, coder inter‐reliability will be assessed as Pearson's *r*. In all instances in which the coding of effect sizes differs between the coders, the coders will re‐extract the relevant data and coding will be conducted jointly. Similarly, the coding of study‐level characteristics will be coded by the same two coders. Identified differences will be rectified through a joint re‐coding.

In addition to effect sizes, we will also code a number of study‐level characteristics. Study‐level characteristics could potentially impact heterogeneity and thereby affect the results or the way in which they should be interpreted. Where possible, we will examine the effects of study‐level characteristics through the use of meta‐regression. Since there is no consensus as to the minimum number of studies needed for conducting such analysis, we will adopt a minimalist's approach in which the minimum number of studies needed for examining a continuous variable is 6 (Fu et al., 2011) and 2 studies per category for categorical variables (Marino et al., 2018). For categorical variables, moderator analysis will be carried out for factors found to have a statistically significant effect in the meta‐regression analyses.

In cases where there is an insufficient number of studies to conduct such analysis, relevant observations concerning the effects of study‐level characteristics will be discussed in the review's discussion. The study‐level characteristics that will be coded are detailed in Table 4.

**Table 4:** Study‐level characteristics

Variable	Description
Effect size derivation	Categorical: Effect size from bivariate or partial effect size
Age	Continuous: Mean age of the sample
Gender composition	Continuous: Proportion of the sample that is male
Year	Continuous: Last year of data collection
Ideology	Categorical: Right‐wing, left‐wing, religious, etc.
Region	Categorical: US, EU, other
Measurement	Continuous: The number of components in a measurement
Publication status	Dichotomous: Published/un‐published
Sample type	Categorical: Random/representative/convenience etc.
Language	Categorical: Language that the publication was written in
Study design	Categorical: Cross‐sectional, longitudinal, case‐control etc.

#### 3.3.5 Assessment of risk of bias in included studies

While the review sets few limitations on inclusion based on study quality, a number of measures of study quality will be coded as study‐level characteristics in order to assess risk of bias. Where possible, we will attempt to analyse how these factors may impact the results through the use of meta‐regression. Where this is not possible, the risk of bias elements of studies will be raised in the discussion of the results. For observational studies, we will carry out the assessment of risk based on the most relevant items derived from the ROBINS‐E (Risk‐of‐bias in non‐randomized studies of exposure) assessment tool. The selected risk of bias items that will be assessed are listed in Table 5.

**Table 5:** Risk of bias measures for observational studies

Variable	Description
Dependent variable(s)	Was the dependent variable measures using a validated instrument?
Independent variable(s)	Was exposure to the independent variable measured using a validated instrument?
Measurement source	Was the source of the measurement for the independent/dependent variable based on self‐reported, clinician reported, administrative reported, or other type of data?
Sampling methodology	Did the study use Systematic, stratified, convenience, quota, purposive, snowball, or another type of sampling?
Temporal ordering	Can temporal ordering be established between the independent and dependent variable(s)?
Analytical rigour	Did the statistical models used adequately control for key confounding variables?
Missing data	Was missing data an issue in the study, and if so, how was it dealt with?
Results	Were results reported in a non‐biased way (e.g., were non‐significant results also reported)?
Reporting bias	Does the study indicate that it has the data to report on a key relationship but fails to do so?
Outcome bias	Does the study indicate that it has the data to report on a relationships with an alternative outcome but fails to do so?

For randomized experimental studies, we will utilize an adapted version of Cochrane's Risk of Bias tool for randomized trials (RoB2). As with the observational studies, risk of bias relates to both the study and outcome levels, but there are two additional risk domains that differentiate experimental from non‐experimental studies, namely: Domain 1 (risk of bias arising from the randomization or other selection process) and Domain 2 (risk of bias due to deviations from the intended interventions). The other domains of the RoB2 tool are analogous to the domains for the observational studies, namely: Domain 3: Risk of bias due to missing outcome data; Domain 4: Risk of bias in measurement of the outcome, and; Domain 5 (risk of bias in the selection of the reported result). Given the nature of the literature, certain items in the tool will not be relevant and as such, a more compact version of the tool will be adopted (e.g., Lum et al., 2020). The following items from RoB2 will be included (Table 6).

**Table 6:** Risk of bias measures for experimental studies

Item	Description
1.1	Was the allocation sequence random?
1.3	Did baseline differences between intervention groups suggest a problem with the randomization process?
2.6	Was an appropriate analysis used to estimate the effect of assignment to intervention?
2.7	Was there potential for a substantial impact (on the result) of the failure to analyse participants in the group to which they were randomized?
5.1	Were the data that produced this result [the results for this study] analysed in accordance with a pre‐specified analysis plan that was finalized before unblinded outcome data were available for analysis?
5.2	Is (Are) the numerical result [results] being assessed likely to have been selected, on the basis of the results, from multiple outcome measurements?
5.3	Is (Are) the numerical result [results] being assessed likely to have been selected, on the basis of the results, from multiple analyses of the data?

Risk of bias coding and assessments will be distributed between two reviewers. A sub‐set of 10% of the studies will be distributed to both reviewers and assessed for inter‐coder reliability.

#### 3.3.6 Measures of treatment effect

There is a lack of consensus as to whether meta‐analysis based on observational studies should give preference to effect sizes derived from bivariate correlations, or correlations derived from the results of multivariate regression models that have controlled for confounders. While standardized coefficients derived from multivariate models may produce estimates closer to the “true” value, between‐study variations in model specification make the synthesizing of these effect sizes quite unbalanced (Hanushek & Jackson, 1977; Hunter & Schmidt, 2004). For this reason, many researchers give preference to bivariate correlations, which offer a consistent and uncontaminated measure across studies (Pratt et al., 2014). The decision whether to use bivariate, multivariate, or a combination of these effect sizes also depends on a study's objectives (Aloe et al., 2016). One of our objectives is to identify the relative magnitude of the effects for different media‐related factors, and bivariate correlations provide more stable estimates for developing a rank‐order of estimates among multiple factors (Hedges & Olkin, 1985). As such, we follow the approach that gives preference to bivariate correlations, but allows for the inclusion of effect sizes standardized from regression models as supplementary effect sizes when that is all that is available. We will use moderator analyses to assess and report the effects of this approach (Aloe & Thompson, Aloe et al., 2016). We believe that this approach is the one most suited to the current review. It is also in line with related works that have been conducted (e.g., Assink, 2017; Najaka et al., 2001; Wong et al., 2010), and serves to enable the inclusion of the greatest possible number of effect sizes (Borenstein et al., 2011).

All effect sizes will be standardized as Fisher's *Z* transformed variables (Borenstein et al., 2009; Rosenthal, 1984), which will be used for reporting of results. Effect sizes will be derived from bivariate correlations, which will be obtained from correlation matrices, or calculated from descriptive data such as means and standard deviations, *t* tests, *χ* ^2^, ANOVAs, and other classical hypothesis tests. The calculation of bivariate effect sizes from descriptive data will be made using the formulas and conventions of Lipsey and Wilson (2001), and the “Practical Meta‐Analysis Effect Size Calculator” available through the Campbell Collaboration website.

For effect sizes derived from bivariate sources, standard errors will be calculated based on the variance of the *z* transformed variable (1/*n* − 3), in which the standard error is calculated as being the square root of the variance.

Where a study reports only the results from linear regression models where the independent variable (IV) and dependent variable (DV) are both continuous we calculate *r* as

$$r=\frac{SD_{x}B}{SD_{y}}.$$

In situations in which standard deviations (SD) are not reported, or when the IV is dichotomous and the DV is continuous, or where the IV is ordinal or continuous and the DV is dichotomous, *r* will be calculated based on the *t* ratio (*B*/SE):

$$r=t/\sqrt{t^{2}+}n-2.$$

In instances in which both IV and DV are dichotomous, and only *B* is reported we will first calculate Cohen's *d* and then convert this to *r* as follows:

$$d=B\left(\frac{\sqrt{3}}{\pi }\right),$$

$$r=\frac{d}{\sqrt{4+d^{2}}}.$$

Where only the odds ratio (Exp. *B*) is reported, *r* will be calculated as

$$r=\frac{\mathrm{OR}\frac{1}{2}-1}{\mathrm{OR}\frac{1}{2}+1}.$$

Standard errors will be calculated, whenever possible, based on a rescaling of the model‐based standard error, which is calculated as

$${\mathrm{se}}_{r}=\frac{r\times {\mathrm{SE}}_{B}}{B}.$$

When the SE is not reported, it may be calculated from reported confidence intervals. In the case of 95% confidence intervals, the SE will be calculated as

$$\mathrm{SE}=\frac{{\mathrm{CI}}_{\text{upper}}-{\mathrm{CI}}_{\text{lower}}}{1.96}.$$

For experimental studies, while it is common to calculate the standard mean difference, Cohen's *d*, there is a tendency for overestimation of effects in small samples (Hedges, 1981). As such, we will use Hedge's *g*, which corrects for this bias. In order to calculate Hedge's *g* we first calculate the correction factor, *j* as

$$J=1-\frac{3}{4\mathrm{df}-1}.$$

With Cohen's *d* already having been calculated based on the means and standard deviations of the groups or other summary statistics, we calculate *g* = *J* × *d*. Subsequently, we calculate the standard error as the square root of *V*_g._, with *V*_g_ calculated as

$$V_{g}=J^{2}xV_{d}.$$

#### 3.3.7 Dealing with missing data

When a study is missing information pertaining to study‐level characteristics or effect sizes, the following actions will be taken in the order in which they appear:

1. Search for supplementary materials
2. Search for access to the original data and replicate the model
3. Search for other studies by the authors that may use the same data
4. Search for studies by other authors that may use the same data
5. Contact the authors with a request to provide the missing data

When a study reports on the effect of a particular factor as not having been statistically significant and no additional information is given or has been acquired through the above steps, an effect size of zero will be entered into the meta‐analysis. While this approach is known to give rise to the potential for an underestimation of the magnitude of the estimate (Durlak & Lipsey, 1991), it is widely considered to be the preferred option over excluding the effect size, which can lead to a possible overestimation of the magnitude of the estimate (Rosenthal, 1995).

If a study presents quantitative findings that are unable to be synthesized in the meta‐analysis, because the statistic presented does not enable conversion or standardization, or there is not enough information available, we will contact the authors to request additional information for calculating the effect size. If such information is not forthcoming, the result may still be included in the discussion of the results of the meta‐analysis, provided that all other inclusion criteria are met.

#### 3.3.8 Assessment of heterogeneity

Heterogeneity will be assessed using Cochran's *Q* and its associated *p* value, as well as the *I* ^2^ statistics. *I* ^2^ scores of >75 indicate high, >50 moderate, >25 medium heterogeneity, and >0 low heterogeneity. When *I* ^2^ = 0 it indicates an absence of heterogeneity.

#### 3.3.9 Assessment of reporting biases

##### Between studies

Reporting bias is a known issue with meta‐analytic studies. Commonly referred to as the “file‐drawer problem”, it is widely understood that researchers have a tendency to avoid publishing non‐significant results, or occasionally results with depicting exceptionally small effects. As a result, the identifiable literature may not be representative of all the studies that have been conducted on a given issue, and the results from pooling these studies may overestimate the true effect size (Rosenthal, 1979). In this review, two methods will be used in order to assess reporting bias.

First, for all analyses including three or more effect sizes, Rosenthal's (1979) Fail‐Safe *N* test will be used in order to identify the number of non‐significant effect sizes that are estimated to be needed in order to bring the pooled estimate below the level of statistical significance. We will follow the rule of thumb approach in which the Fail‐Safe *N* is equal to *k* × 5 + 10, with *k* being the number of effect sizes in the analysis. When the number of missing studies is larger than the Fail‐Safe *N*, it indicates that the estimate is robust against reporting bias.

Second, we will use the Trim‐and‐fill method (Duval, 2005; Duval & Tweedie, 2000a, 2000b). This method estimates the number of studies missing on the extremities of a funnel plot and augments the observed data in order to create a more symmetric distribution. Based on this, adjusted estimates and heterogeneity statistics are generated and enable the assessment of the degree to which the results are sensitive to reporting bias.

#### 3.3.10 Within studies

A common issue in different literatures concerns outcome reporting bias, in which studies may not report on the relationship between variables and certain outcomes even when it is clear that they have enough information to report on such relationships. In some cases, a study may make no mention of possible alternative outcomes, even though the researchers have collected data pertaining to it. In order to assess and account for outcome reporting bias, we will search for online supplementary materials and open access data for all included studies in order to identify any additional sources of reporting bias as they pertain to unreported outcomes. For studies for which supplementary materials or original data is found, we will code and report as to whether such materials provide evidence to support the identification of either of the two types of reporting bias described above.

#### 3.3.11 Data synthesis

Meta‐analysis will be conducted for each factor for which at least two unique effect sizes are found. We will use Biostat's Comprehensive Meta‐Analysis (CMA) software (Borenstein et al., 2009) to perform all analyses, including tests for publication bias, meta‐regressions, and moderator analyses. Given the expected nature of the data, it is anticipated that all analyses will use Random effects. We will utilize the random effects estimator for *τ*2, the between‐study variance, that is pre‐programmed in CMA V3, which is the Method of Moments approach of DerSimonian and Laird (1986).

#### 3.3.12 Subgroup analyses and investigation of heterogeneity

In order to further investigate and understands sources of heterogeneity, and to identify ways in which estimates for different factors may fluctuate between different contexts and conditions, a number of additional analyses are planned.

Firstly, when the studies included in a given analysis provide sufficient information, subgroup analysis will be performed based on any of the following sub‐groupings within studies:

1. Age grouping
2. Gender
3. Ideology/religion/ethnicity
4. Country/region
5. Different groupings on categorical variables (e.g. low/medium/high education, socio‐economic status, etc.).
6. Different measurements of outcome(s)/Different outcomes

As described above, there are a number of study‐level characteristics upon which we will perform Meta‐regression analyses. These analyses will be used to assess the effects of the following factors on pooled estimates:

- Region from which the sample was derived (EU, United States, and other)
- Ideological strain examined by the study (non‐specific/mixed ideologies, right‐wing, left‐wing, Islamist, and other (which included separatist and ethno‐nationalist).
- Year of data collection
- Mean age of study sample
- Proportion of males in study sample
- Different measures of outcomes (e.g., dichotomous, ordinal, etc.)
- Different types of studies (e.g., cross‐sectional, longitudinal, case‐control).

We take a minimalist approach in which a minimum of two studies from each category is needed in order to perform meta‐regressions for categorical variables, and a minimum of six studies is needed for continuous variables. For categorical variables, wherever the meta‐regression provides a statistically significant effect (*p* < .10), moderator analyses will be used to assess the degree of between category heterogeneity and differences in the estimates.

As our approach is to identify and utilize bivariate correlations as our preferred effect size, and only utilize effect sizes derived from multivariate models when bivariate calculations are not possible, we will not assess differences in the effect sizes from different studies based on the other (both included and excluded) covariates in a study. However, for any analysis for any particular factor that includes at least two effect sizes derived from different sources (bivariate or standardized partial effect sizes), we will assess the impact of combining the effect sizes through meta‐regression. As per the above‐noted approach, if the meta‐regression indicates a statistically significant effect, we will subsequently carry out a moderator analysis to identify the degree of between category heterogeneity and differences in the estimates across the categories.

#### 3.3.13 Sensitivity analysis

Sensitivity analysis will be carried out for each analysis that includes three or more studies by using the “leave‐one‐out” method. In this method, the meta‐analysis is iteratively repeated *k* times (*K* = the number of studies), with a different study excluded at each iteration and the estimates and heterogeneity statistics re‐calculated. The results provide for the ability to examine whether a specific study is responsible for greatly influencing the results (Viechtbauer & Cheung, 2010). We will report on those analyses in which the removal of a single study was found to reduce heterogeneity by at least one level, with the levels being low (*I* ^2^ < 25), moderate (*I* ^2^ < 50), high (*I* ^2^ < 75), and very high (*I* ^2^ > 75).

### 3.4 Treatment of qualitative research

We do not plan to include qualitative research.

## ROLES AND RESPONSIBILITIES

- Content: Michael Wolfowicz and Badi Hasisi
- Systematic review methods: Michael Wolfowicz and David Weisburd
- Statistical analysis: Michael Wolfowicz and David Weisburd
- Information retrieval: Michael Wolfowicz

## SOURCES OF SUPPORT

This study was supported by the Horizon 2020 Grant 699824, DHS Science and Technology Directorate and the Five Research and Development (5RD) Countering Violent Extremism Network, provided to the Campbell Collaboration.

## CONFLICT OF INTERESTS

The authors have been involved in the development of one study that is expected to be included in the review.

## PRELIMINARY TIMEFRAME

The systematic review is set to be submitted by the end of 2021.

## PLANS FOR UPDATING THE REVIEW

New searches are set to be conducted for an updated review in 2022, with a view to having an updated review completed by 2024.
